# Active infection with *Onchocerca volvulus* and the linkage to epilepsy/nodding syndrome

**DOI:** 10.1371/journal.pntd.0012076

**Published:** 2024-05-09

**Authors:** Kathrin Arndts, Josua Kegele, Manuel Ritter, Clarissa Prazeres da Costa, Achim Hoerauf, Andrea S. Winkler

**Affiliations:** 1 Institute of Medical Microbiology, Immunology and Parasitology (IMMIP), University Hospital Bonn (UKB), Bonn, Germany; 2 German-West African Centre for Global Health and Pandemic Prevention (G-WAC), Partner Site Bonn, Bonn, Germany; 3 Hertie Institute for Clinical Brain Research, Department of Neurology and Epileptology, University of Tübingen, Tübingen, Germany; 4 Center for Global Health, School of Medicine and Health, Technical University of Munich, Munich, Germany; 5 Institute for Medical Microbiology, Immunology and Hygiene, TUM School of Medicine and Health, Technical University of Munich, Munich, Germany; 6 German Center for Infection Research (DZIF), Partner Site Munich, Munich, Germany; 7 German Center for Infection Research (DZIF), Partner Site Bonn-Cologne, Bonn, Germany; 8 Department of Neurology, Technical University of Munich, Munich, Germany; 9 Department of Community Medicine and Global Health, Institute of Health and Society, University of Oslo, Oslo, Norway; 10 Department of Global Health and Social Medicine, Harvard Medical School, Boston, Massachusetts, United States of America; Federal University of Agriculture Abeokuta, NIGERIA

We appreciate the comment “Onchocerciasis-associated epilepsy and biomarkers” from Robert Colebunders and colleagues published in PLOS NTD 2024 [1] as response to our original publication “Epilepsy and nodding syndrome in association with an *Onchocerca volvulus* infection drive distinct immune profile patterns”.

In our study [2], we focused on the presence of an **active** *Onchocerca volvulus* (OV) infection, determined by polymerase chain reaction (PCR) from skin snips, in the context of epilepsy/nodding syndrome (NS) and not on the immune profiles of onchocerciasis-associated epilepsy (OAE) patients. Therefore, we did not present the OAE criteria. OAE is still a matter of debate since a causal relationship between OV infection and epilepsy/NS has not been established so far. We would like to emphasize that a positive OV16 test result confirming IgG4 antibodies to the recombinant antigen OV-16 [3], which is part of the OAE core criteria, only indicates exposure/previous infection but does not confirm an **active** infection [4], in contrast to the highly specific and sensitive PCR [5]. Indeed, we observed during our African field studies that most of the individuals, including controls, were antibody-positive in the OV16 test. This was also confirmed by Edridge et al. [6].This is not surprising in a previously OV high-endemic area like Mahenge. Thus, this test cannot not discriminate between individuals who developed epilepsy, NS or remained healthy. Contrary to the application of the OAE criteria [7–17], studies investigating epilepsy/NS in context of an **active** OV infection are scarce. It has been shown that immunity and metabolism are strongly modulated in individuals who are actively infected with filarial nematodes (or helminths) in comparison to individuals that have cleared the infection [18–20]. To find out more about the possible ways of immunomodulation of OV and its effect on neurological signs/symptoms, we also suggested in our discussion that future studies should take into account the presence of patent and latent infections, which unfortunately was not possible in our study due to the small group sizes.

We fully agree with the suggestion of Colebunders et al. [1] to include an additional control group, namely OV-infected individuals without any epilepsy, in future studies to confirm our findings. Indeed, we clearly mentioned this as a limitation of our study. Most of the individuals with epilepsy/NS were already known and registered patients at the Mahenge Epilepsy Clinic, where they received medication. Nevertheless, we initially had five individuals without epilepsy/NS (caregivers, family members of patients) who were positive for OV determined by PCR from their skin snips. Unfortunately, they did not provide urine and blood samples and were therefore excluded from the study.

The overall conclusion from our publication was that biomarkers *might be useful* to discriminate epilepsy from NS in individuals with or without an OV infection, which was met with criticism by Colebunders et al. [1]. One of the potential candidates could be N-acetyltyramine-O, β-glucuronide (NATOG), which has been previously described as a potential non-invasive biomarker of active *O*. *volvulus* infections and which was significantly elevated in NS patients with OV infection compared to epilepsy patients without OV infection and to controls in our study. We drew our conclusions carefully and did not state that NATOG had discriminative power pointing to further limitations (e.g., small sample size, lacking OV-infected individuals without epilepsy/NS, further studies needed to proof that NATOG might be a potential biomarker) of our study. We are fully aware that the described method of NATOG measurement is not suitable so far for the application in the field due its technical complexity and thus argued that a simplified test version, e.g., a rapid test, could support a diagnosis of **active** OV infection in the field, particularly in children that are reluctant to undergo invasive methods. Colebunders et al. [1] continued to argue that “While determining NATOG levels may be useful in persons with epilepsy in high OV transmission zones, the NATOG test will have low discriminating power to differentiate between NS and other forms of epilepsy because most other forms of epilepsy in such areas will be OAE.” This statement is incorrect as epilepsy has many different causes, also in endemic areas where the OV infection rate is high.

In summary, our study focused on **active** OV infection in people with epilepsy/NS and not on people with OAE. We cautiously stated our conclusions regarding potential biomarkers to differentiate people with epilepsy and those with NS with or without an active OV infection and call for further studies. Whether or not OV is the causal agent for epilepsy or NS in OV-endemic areas is not yet conclusively established and may require prospective studies comparing different areas with long-term follow-up of healthy individuals and those who eventually develop epilepsy/NS.
